# News and Miscellany

**Published:** 1883-04

**Authors:** 


### NEWS AND MISCELLANY.

New Veterinary Schools.—The Russian Government has instituted a new Veterinary School at Lemberg, Poland. Dr. Leifmann, of the Kazan Veterinary School, has been appointed Director, and Drs. Baranski and Kaydi Professors.—*Veterinary Journal.*

Ten thousand dollars have been subscribed by Mr. Gillingham towards the endowment of a Veterinary School in connection with the University of Pennsylvania. A similar sum was previously contributed by Mr. J. B. Lippincott.

The Number of Veterinary Surgeons in this Country, according to the last census, is 2,130· Of these 1,457 are natives of the United States, 104 of Ireland, 156 of Germany, 252 of Great Britain, and 171 of other countries. According to a register prepared with much care by the *Journal of Comparative Medicine* only about one-half of these have degrees.

Bitten by a Squirrel.—Dr. W. G. Stedman, of Southington, Conn., was bitten last week in the hand by a pet squirrel; severe lymphangitis ensued, and it was feared he would lose his arm.—*Medical Record.*

Cold Kills Trachinæ.—According to the experiments of Bonley and Gibdir, a temperature of 12° to 15° C. for five hours will destroy trachinæ.—*Chicago Medical Review.*

A Contrivance to Prevent "Balling" of Horses Feet.—The English *Live Stock Journal* gives an accouut of a contrivance adopted by Col. Thompson, of the Fife Hounds, for the protection of the horse's feet from snow, from which veterinarians may take a hint. Col. Thompson takes a sheet of gutta-percha about a quarter of an inch thick, cuts out a set of plates a little larger than the hoof inside the shoe, softens them in hot water, and moulds them inside the shoe over the frog, so that a face of gutta percha touches the ice and snow, and "balling" with snow impossible. These plates remain until the end of bad weather.

Small-Pox in Birds.—Dr. William Gayton, Medical Superintendent of the Small-Pox Hospital at Homerton, England, writes (*British Medical Journal*) concerning this subject as follows:

"Apropos of 'small-pox in birds,' I may, perhaps, mention the fact that some years ago a former steward of this hospital was in the habit of breeding a large number of canaries. As these arrived at maturity it was a common occurrence to find many of them dead, and presenting evidence of having suffered from some eruptive disease. It was further observed that, when the hospital contained a somewhat large number of patients, the mortality among the birds increased, and *vice versa.*"

Phthisis Conveyed from Dogs to Man.—Dr. E. G. Janeway relates a number of cases of phthisis (*Archives of Medicine*) illustrating its possible contagiousness. Among others was the case of a phthisical young man who kept a pet dog. He was accustomed to sleep with the dog nestling in his arms. The animal became affected with a cough and subsequently died. Another dog shared the same fate; a third dog suffered from a cough, but its owner died of phthisis and the dog subsequently recovered.

LOSS OF CUD.—Loss of cud or suspension of rumination in cows is due to indigestion, from inaction of the muscular coats of the stomach. The remedy is to clear the bowels and stomach of the gathered indigested matter by a brisk purgative; for instance, a quart of linseed oil or twenty ounces of Epsom salts, and then to give some easily digested and laxative food, as bran mashes or linseed meal steeped in water twelve hours. On the first appearance of the suspension of rumination this course should be pursued, as it is usually due to overfeeding, and the bowels require to be relieved before any change can be procured, Continued loss of cud ends in impaction of the stomach with disorder of the liver and kidneys and a probable serious attack of fever.

ONTARIO VETERINARY SOCIETY.—The fall term examinations of the Ontario Veterinary College, Toronto, were concluded Dec. 21st, 1882, when the following students passed a highly creditable examination, and were duly awarded the diploma of the council: C. W. Stone, Detroit, Mich.; H. G. Marshall, Dungannon, Ireland; S. G. Reed, Rushsylvania, Ohio; F. Fisher, Baillieboro, Ont.; W. F. Kidd, Cookstown, Ont.; Ward Woodhull, Angola, Ind.; H. H. Clement, Coldwater, Mich.; George E. Ferling, Indianapolis, Ind.; I. N. Perdue, Wingham, Ont. C. W. Stone, H. H. Clement and I. N. Perdue were awarded honors.

A REPORT OF THE TREASURY CATTLE COMMISSION, which was sent to the House recently, describes the sites selected for quarantine stations for imported cattle at Portland, Boston, New York and Baltimore. The report says that "it is vain to hope that England will remove the restrictions imposed so long as we fail to show that the last vestige of infection has been wiped out from our land," and, further, "that nothing short of the absolute and undeniable extinction of this disease in the United States will reopen the British market to our live cattle, and save us those millions that we are now every year prodigally, and we might almost say insanely, throwing away." The commission estimates the sum required to stamp out the lung plague at $2,000,000, and recommends the requisite legislation, The governing principle in all their recommendations is that the Federal Government shall forbid the movement of store cattle out of any infected State, Territory, or district except after a quarantine such as is now imposed on the cattle imported from infected foreign countries.

A HOSPITAL FOR ANIMALS.—The Department of Agriculture has leased a piece of ground near the boundary line of the north-eastern section of the city, to be used as an experimental farm and hospital in connection with investigations of diseases of animals. The grounds are being put in order and buildings erected thereon. Dr. D. E. Salmon, who has for several years been employed by the department in the investigation of diseases of cattle, swine and poultry, will arrive in Washington about May 1st to take charge of the work. Dr. Salmon will bring with him a number of cattle and sheep, and the experiments will begin soon after his arrival. The Pasteur system of inoculation will be adopted, with such additions and qualifications as have been suggested by Dr. Salmon's own discoveries while engaged in investigation at his farm near Asheville, N. C. The investigations now to be made will be on a much larger scale than any heretofore attempted by the department, and will be conducted with the view of ascertaining the origin, causes and nature of the Texas cattle fever, pleuro-pneumonia, and hog and chicken cholera, together with means of preventing and curing these diseases.

TABLES IN COMPARATIVE PHYSIOLOGY.—We call the attention of our readers to the very valuable compilation of facts in Comparative Physiology found elsewhere in the JOURNAL. It is a noteworthy fact that hitherto if a person wished to know the pulse, temperature, respiration, urine amounts, &c., of the domestic animals, the facts could not be obtained except by consulting works in German or French. Not long ago a whole series of experiments was vitiated because the experimenter did not know the normal temperature of the rabbit. Most of the important facts needed for practical reference are embodied in the tables now published. These tables will be sold separately by the Publisher, Mr. W. R. Jenkins. Price, 10 cents.

THE VIVISECTION QUESTION commands much attention abroad. In Sweden the Government has decided not to prohibit vivisection in that country, in spite of the appeal made to them by the Diet in reference thereto last session. In Denmark the Society for the Protection of Animals at Copenhagen, which is under the patronage of the King of Denmark, offers two prizes, one equivalent to £40 and the other to £80, for the best essays upon the possibility of replacing vivisection in physiological research by experiments upon the bodies of animals recently killed.

A HORSE CALLS ON A DOCTOR.—A reliable friend tells us about a horse that felt sick in the night, and, breaking out of its stable, made its way to the stable of a veterinary surgeon who had before treated the animal for sickness. It stood there several hours awaiting the opening of the stable in the morning, and when admitted soon fell dead.—*Saginaw Courier.*

INTELLIGENCE IN ANIMALS.—Mr. Romanes remarks in his book that there are few recorded instances of intelligence in bears ; the following facts may therefore be worth recording: In the Clifton Zoological Gardens there are two female polar bears, between 2½ and 3 years. which came here quite young. One of these shows remarkable intelligence in cracking cocoa-nuts. A nut was thrown in the tank; it sank a long way. and the bear waited quietly till after some time it rose a little out of her reach. She then made a current in the water with her paw, and thus brought it within reach. This habit has already been several times noticed in polar bears. She then took it on shore and tried to break it by leaning her weight on it with one paw. Failing in this, she took the nut between her fore paws, raised herself on her hind legs to her full height. and threw the nut forward against the bars of the den. three or four feet off. She then again leaned her weight on it, hoping she had cracked it, but failed again. She then repeated the process, this time successfully. The keeper told me she employed the same method to break the leg-bone of a horse. That this is the result of individual experience, and not of instinct, is clear from the fact that her companion has not learned the trick of opening them thus, nor could this one do it when she first came. The method of throwing it is precisely similar to that adopted by the Cebus monkey described by Mr. Romanes.—*Nature.*

A CASE OF FILARIA OCULI OCCURRING IN BOTH EYES OF A HORSE.—A five year old horse was found to have a worm in the left eye. This was successfully removed, and almost before the eye had recovered a worm was discovered in the opposite one. This was also successfully operated on. This case is worthy of record only on account of the occurrence of a worm in both eyes. I have never heard of a similar case. The patient did well, and there was no ill effects.—*Quarterly Journal of Veterinary Science in India.*

BACTERIA having been found to be the fruitful cause of many diseases, M. Martineau now claims to have discovered that of syphilis. He made some experiments by cutting out a hard chancre and immersing it in culture bouillon of Pasteur. In this bacteria developed, and some of it was injected into the tissues of a pig near the penis. By the next day similar bacteria were found in the blood of the animal, and in a month there was a syphilitic eruption on the skin, with falling out of the hair. He then took some of the serum from another chancre and inoculated a pig with it. On examining the blood four days after, bacteria like those in the first experiment were found and in fourteen days a syphilitic papular eruption appeared on the skin. The bacteria found in the blood of these two pigs were then cultivated, and some of the fluid injected underneath the skin of a pig and a kid. Repeated examinations of the blood of these animals have failed to show the presence of bacteria, showing that they cannot be transmitted from one animal to another.—*Weekly Medical Review.*

